# Functional protein representations from biological networks enable diverse cross-species inference

**DOI:** 10.1093/nar/gkz132

**Published:** 2019-03-08

**Authors:** Jason Fan, Anthony Cannistra, Inbar Fried, Tim Lim, Thomas Schaffner, Mark Crovella, Benjamin Hescott, Mark D M Leiserson

**Affiliations:** 1 Department of Computer Science and Center for Bioinformatics and Computational Biology, University of Maryland, College Park, USA; 2 Department of Biology, University of Washington, USA; 3 University of North Carolina Medical School, USA; 4 Department of Computer Science, Boston University, USA; 5 Department of Computer Science, Princeton University, USA; 6 College of Computer and Information Science, Northeastern University, USA

## Abstract

Transferring knowledge between species is key for many biological applications, but is complicated by divergent and convergent evolution. Many current approaches for this problem leverage sequence and interaction network data to transfer knowledge across species, exemplified by network alignment methods. While these techniques do well, they are limited in scope, creating metrics to address one specific problem or task. We take a different approach by creating an environment where multiple knowledge transfer tasks can be performed using the same protein representations. Specifically, our kernel-based method, MUNK, integrates sequence and network structure to create *functional protein representations*, embedding proteins from different species in the same vector space. First we show proteins in different species that are close in MUNK-space are functionally similar. Next, we use these representations to share knowledge of synthetic lethal interactions *between species*. Importantly, we find that the results using MUNK-representations are at least as accurate as existing algorithms for these tasks. Finally, we generalize the notion of a phenolog (‘orthologous phenotype’) to use functionally similar proteins (i.e. those with similar representations). We demonstrate the utility of this broadened notion by using it to identify known phenologs and novel non-obvious ones supported by current research.

## INTRODUCTION

A primary challenge of research with model organisms is to transfer knowledge of genetics—i.e. a mapping of genotype to phenotype—between model organisms and humans. The main promise of researching model organisms stems from researchers’ ability to measure the organisms in ways that are infeasible in humans. To realize the promise of this research, it is crucial to transfer knowledge between species—ideally, in two directions. First, discoveries in model organisms can be transferred to improve knowledge of human genetics (e.g. via homology). Second, knowledge of human genetics can be transferred to design better experiments in model organisms (e.g. for disease models).

More specifically, cross-species knowledge transfer can enable a wide variety of applications. First and foremost is the large-scale annotation of protein function by transferring function annotations (e.g. from the Gene Ontology (1)). Addressing this problem remains valuable, even in the era of high-throughput genomics, as fewer than 1% of protein sequences in UniProt have experimentally-derived functional annotations (2). Another application of cross-species knowledge transfer is for *pairwise* gene function (genetic interactions). Knowledge of synthetic lethal genetic interactions is crucial for the study of functional genomics and disease (3,4). Since genome-wide measurement of synthetic lethal interactions in humans is currently infeasible, computationally transferring knowledge of these interactions from model organisms (such as yeast or mouse) to humans (and human cancers) has become a focus of recent research. A third but less well-explored application is in predicting human disease models through ‘orthologous phenotypes’ or phenologs (5). McGary *et al.* (5) reasoned that while conserved genes may retain their *molecular* functions across species, conserved molecular function may manifest as different ‘species-level’ phenotypes. As such, they introduced a statistical test to identify such phenologs.

Cross-species knowledge transfer is quite challenging because many model organisms diverged from humans millions of years ago and have fundamentally different genetic architectures. In many cases, only a relatively small subset of genes between species have sequence homologs. Further, as species diverge, protein functions change and are re-purposed (e.g. (6)) through divergent and convergent evolution, and genetic interactions are often rewired (7,8).

Existing computational approaches to transfer knowledge across species rely on matching a subset of genes (proteins) in different species by heredity (genetic orthology) or function (functional orthology). One class of computational approach uses sequence data to match genes (proteins) (9,10). A second class of methods expands beyond sequence by using proteomics data to match proteins, through protein structure prediction (e.g. (11)) or alignment of protein–protein interaction networks (12–17), commonly called the network alignment problem.

Many cross-species biological problems cannot be formulated as a matching problem; for example, genetic interactions and phenologs are fundamentally measures of *sets* of genes. This motivates the idea of creating general-purpose multi-species protein representations. These in turn could be used to generate a matching, but could also be interpreted as a vector space or used as input to a learning algorithm. General-purpose representations are fast becoming adopted in different areas of machine learning, from natural language processing (e.g. (18)) to network science (e.g. (19)), and recently have begun to be adopted for biological networks (20,21).

However, the problem of learning multi-species protein representations from network and sequence data remains largely unexplored. Jacunski *et al.* (22) showed that protein representations derived from graph theoretic measures of network structure can be used to transfer knowledge of synthetic lethal interactions across species. However, their approach creates the representations in each network independently, does not use sequence data at all, and uses a set of handcrafted features chosen for a particular task. Gligorijević *et al.* (23) use matrix factorization based on sequence similarity with PPI-based Laplacian smoothing to cluster cross-species protein pairs. However that method does not embed nodes in a common vector space, instead computing scores for a subset of protein pairs that are used as an input to max-weight matching for network alignment. More recently, Khurana *et al.* (24) developed an embedding for proteins in multiple species for an application concerning neurodegenerative diseases.

### Contributions

In this work, we address the limitations of task-specific protein representations, in a way that allows us to move beyond simple matching of proteins across species. We combine protein–protein interaction networks and sequence data from multiple species into unified, biologically meaningful protein representations using network diffusion. Network diffusion is a natural tool for capturing aspects of local and global network structure that correlate with functional similarity of nodes (25). The key insight of our approach is that homologous proteins can serve as landmarks for relating proteins in different species. We then show the similarity scores derived from these representations as well as the representations themselves are useful for distinct tasks.

Our method makes only two assumptions. First, it assumes that protein function can be captured using a similarity score that is a *kernel*, which encompasses a broad class of useful metrics. Second, it assumes that sequence homology is known for some subset of landmark proteins across the different species.

In this paper, we use a *diffusion* kernel to create functional protein representations and call the resulting method MUNK (MUlti-Species Network Kernel). We then evaluate the MUNK protein representations on three multi-species tasks.

1. **Multi-species functional similarity**. We show that cross-species matchings and similarity scores derived from the MUNK representations are significantly correlated with cross-species protein function, and achieve comparable performance to two existing network alignment matching methods. We also perform a proof-of-concept experiment using MUNK to predict protein function in humans by simultaneously leveraging networks from humans and multiple model organisms.
2. **Multi-species synthetic lethality**. We train classifiers on MUNK-representations for *pairs* of genes in order to predict synthetic lethal interactions (SLI) in multiple species. We find that classifiers accurately identify SLI in multiple species *simultaneously*, and that they achieve comparable performance to the SINaTRA algorithm (22).
3. **Phenologs (orthologous phenotypes)**. We generalize the notion of orthologous phenotypes beyond evolutionarily conserved sequence with a broader definition of functional similarity. We then identify phenologs between human and mouse that are statistically significant for this broader similarity level. A subset of our predicted phenologs match those identified in (5); additionally, we also predict many new phenologs and support these new predictions with biological literature.

Together, these tasks encompass transferring knowledge both between model organisms, and between model organisms and humans.

## MATERIALS AND METHODS

**Figure 1.**
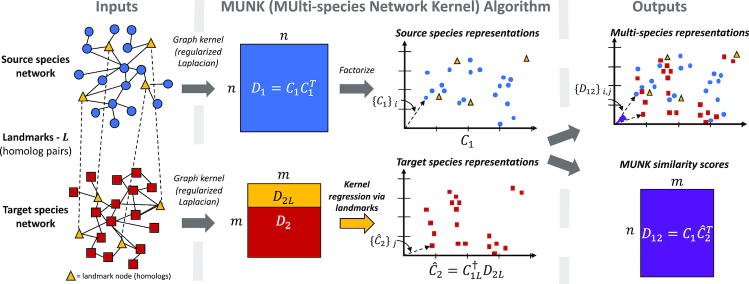
Given a source PPI network, a target PPI network, and a set of landmark (homolog) pairs across species, MUNK computes diffusion kernels for each network. Then, MUNK factorizes the diffusion kernel for the source species into its reproducing kernel Hilbert space (RKHS). Finally, MUNK solves a linear system of the source species’ RKHS and the target species’ diffusion kernel to create a multi-species vector embedding of source and target proteins. The inner products of these embeddings correlate with functional similarities and the embeddings themselves allow for functional comparisons between proteins across the two networks.

The central contribution we make in this paper is to introduce *network-based functional representations for proteins in different species*. MUNK, as shown in Figure 1, leverages properties of kernel functions as tools for measuring the similarity of nodes in a network and for creating embeddings. While the use of kernels for the study of individual networks is well known (25), it remains an open problem to construct network-based kernels that capture the similarity of nodes between *different* networks. This is the challenge that MUNK addresses.

Starting from a given kernel (node similarity function), and given the PPI networks for a source and a target species, MUNK starts by performing a kernel embedding of the source species nodes (proteins). That is, for each node *v*_*i*_ in the source species, MUNK computes a vector ϕ(*v*_*i*_) such that the vector inner product for any two nodes is equal to the kernel similarity of the nodes. The vectors ϕ(*v*_*i*_) can be thought of as an embedding of the source network into a geometric space.

The key step in MUNK is to also embed the nodes of the target species in the *same* vector space, thus creating unified multi-species protein representations. MUNK does this through the use of landmarks—nodes that are known to be the same in both species. For PPI networks, homologous proteins play the role of landmarks. MUNK then places target nodes in the vector space so as to capture their similarity to the landmark nodes.

The result is a joint embedding of both networks in the same vector space. Because both source and target nodes are placed in a way that captures similarity to the same set of landmarks, it becomes possible to score and estimate similarities between nodes in different species. It is also useful to create representations for pairs of proteins, in which case we simply add together the embeddings of two proteins. More details and rationale are provided below.

While MUNK can be used with any network kernel, in this study, we use the regularized Laplacian network kernel in order to capture functional similarity between proteins in different species. The regularized Laplacian kernel is a natural choice for this task because of its close relationship to the principle of ‘guilt-by-association’ often used by protein function prediction methods (26), and to network diffusion methods (e.g. see (27)).

### Multi-species network kernel (MUNK) embedding

We start by noting that there are a large variety of kernels derived from networks (28) (Ch. 2) and they can model processes such as random walks, heat diffusion, PageRank, electrical resistance, and other ways of capturing node similarity in a network. Many kernels derived from networks have been applied successfully for a wide range of problems associated with biological network analysis (e.g. see review in (25,29)).

Though many previous studies have used graph kernels to compare nodes *within* biological networks to our knowledge few methods have utilized kernels to fulfill the goal of comparing nodes *across* multiple biological networks. To do so, MUNK relies on a basic property of a kernel: any kernel is also an *inner product* in a particular space. That is, for any kernel κ( ·, ·), there is a function ϕ( · ) that assigns vectors to nodes such that that κ(*i, j*) = ϕ(*i*)^*T*^ϕ(*j*). The corresponding vector space (termed the *reproducing kernel Hilbert space* (RKHS)) introduces a geometric interpretation for the kernel function. In the context of a kernel for network nodes, the RKHS representation can be thought of as an *embedding* of the network into a vector space in a manner that captures node similarity via inner product.

Conceptually, MUNK approaches the multi-network challenge by constructing a *joint embedding* of the nodes of two (or more) networks in a *single* RKHS. The key to the MUNK method is that, within this RKHS, the similarity of nodes from *different* networks is still captured by inner product, resulting in a *multi-network kernel*.

Given a *source* network *G*_1_, a *target* network *G*_2_, and a kernel κ, the strategy taken by MUNK is to start by embedding the nodes of the source network *G*_1_ using the associated function, ϕ. As described above, this means that inner product between embedded *G*_1_ nodes will capture similarity as described by κ. Next, MUNK makes use of *landmarks*—pairs of nodes in the source and target networks with identical function. The idea is to embed the nodes of the target network *G*_2_ into the same space as the nodes of the source network *G*_1_, such that their position in that space reproduces their similarity to the landmarks of *G*_2_. Essentially, we posit that locating a node from *G*_2_ based on its similarity to the set of landmarks in *G*_1_ will also establish its similarity with the *non-landmark* nodes in *G*_1_. As a result, MUNK creates a multi-network kernel—a single kernel function that captures both the similarity of nodes to each other in the source network *G*_1_, and the similarity of nodes between the source and target networks *G*_1_ and *G*_2_. This is a fundamentally different strategy than has been used in past manifold-alignment methods (30,31), in which alignment is based on Euclidean distance. Aligning on Euclidean distance does not respect inner product, and so the similarity captured by the kernel is not preserved in the alignment.

We now define the MUNK approach formally. Let the matrix $K \in \mathbb {R}^{n\times n}$ hold the values of the similarity function κ(*i, j*) for all pairs of *n* proteins from a particular species. For any such kernel matrix, we can write *K* = *CC*^*T*^ where *C* is an *n* × *k* matrix, uniquely defined up to an orthogonal transformation, with *k* ≤ *n*. This follows from the fact that *K* is positive semidefinite, and means that $\kappa (i,j) = c_i^Tc_j,$ where *c*_*i*_ is the *i*th row of *C*, represented as a column vector. As explained above, the similarity between nodes *v*_*i*_ and *v*_*j*_ is exactly given by the inner product of their corresponding vectors, *c*_*i*_ and *c*_*j*_.

Now consider a source network *G*_1_ = (*V*_1_, *E*_1_) and a target network *G*_2_ = (*V*_2_, *E*_2_) with |*V*_1_| = *m* and |*V*_2_| = *n*. We assume the existence of some (small set of) nodes that correspond between *G*_1_ and *G*_2_. In the case where *G*_1_ and *G*_2_ are PPI networks, these can be orthologous proteins. For example, for orthologous proteins in different networks, it is well known that evolutionary rates differ over a wide range of magnitudes (32). Some proteins are highly conserved and their orthologs will have substantial sequence similarity between *G*_1_ and *G*_2_. Thus, there is generally a small subset of proteins that can be confidently mapped between *G*_1_ and *G*_2_ based on the magnitude and uniqueness of the similarity of their sequence information which we refer to as landmarks.

We then proceed as follows. First, we construct kernel (similarity) matrices $D_1 \in \mathbb {R}^{m\times m}$ and $D_2\in \mathbb {R}^{n\times n}$ corresponding to *G*_1_ and *G*_2_. Next, we construct RKHS vector representations *C*_1_ for nodes in the source network *G*_1_ from the factorization $D_1 = C_1C_1^T.$ Let *C*_1*L*_ be the subset of the rows of *C*_1_ corresponding to landmarks, and let *D*_2*L*_ be the subset of the rows of *D*_2_ corresponding to landmarks (in corresponding order).

The key step then is to construct the vector representations of the nodes in the target network *G*_2_. To do this, we treat the similarity scores *D*_2*L*_ in the target network as if they applied to the landmarks in the source network *G*_1_. For a given node in the target network, we want to find a vector for the node such that its inner product with each *source* landmark vector is equal to its diffusion score to the corresponding *target* landmark. This implies that the RKHS vectors, $\hat{C}_2$, for nodes in the target network *G*_2_ should satisfy $D_{2L} = C_{1L}\hat{C}_{2}^T$. This underdetermined linear system has solution set,

(1) \begin{equation*} \hat{C}_2^T = C_{1L}^\dagger D_{2L} + (I - C_{1L}^\dagger C_{1L})W, \end{equation*}

where $C_{1L}^\dagger$ is the Moore–Penrose pseudoinverse of *C*_1*L*_, and *W* is an arbitrary matrix. We choose the solution corresponding to *W* = 0, meaning that the vectors $\hat{C}_2^T$ are the solutions having minimum norm.

The resulting solution, $\hat{C}_2$, represents the embedding of the nodes of *G*_2_ (the target) into the same space as the nodes of *G*_1_ (the source). We can then compute similarity scores for all pairs of nodes across the two networks as $D_{12} = C_1\hat{C}_2^T.$ This yields *D*_12_, an *m* × *n* matrix of similarity scores between nodes in the source and target networks.

### MUNK and the regularized Laplacian

While our method can be used with any kernel, in this paper we focus on using a kernel intended to capture functional similarity of proteins. To motivate our choice of kernel function *k*( ·, ·), we consider the function prediction problem on a single network, *G* = (*V, E*), with |*V*| = *n*, where *G* has adjacency matrix *A* with entries *a*_*ij*_. For simplicity we consider *G* to be unweighted, so *a*_*ij*_ ∈ {0, 1}. Extensions of our arguments to weighted graphs are straightforward.

A central idea used throughout network-based functional prediction methods is that of *guilt by association*—that is, two nodes that are near each other are more likely to share the same label than two nodes that are far apart. In the context of protein function prediction, this principle has been well established. For example, the authors in (26) show that two neighbors in the protein interaction network are more likely to have the same function than a randomly chosen pair.

Consider the case of determining whether nodes should receive a particular function label where we label a node with a 1 if it should receive the label and 0 otherwise. We are interested in the case in which the label is rare and we believe that nodes may be mislabeled (e.g. some nodes labeled 0 should actually be labeled 1). We assume that there is some current labeling which is incomplete; that is, most nodes are currently labeled 0, and some nodes labeled 0 should actually be labeled 1. Define the vector *y* such that *y*_*i*_ = 1 if node *v*_*i*_ has label 1, and zero otherwise. The goal of the function prediction problem is to estimate a new $\hat{y}$ that is a better labeling of nodes in *V*.

To address this problem, we proceed as follows (33,34). First, we posit that *y* should not differ too much from $\hat{y}$—nodes should tend to be given the labels they already have. Second, we also posit that neighbors in *G* should tend to be given the same label—this is the guilt by association principle.

Note that these two goals are in conflict: fully following the first principle leaves all labels unchanged, while fully following the second principle makes all labels the same (either 0 or 1). To balance these, we define the following optimization:

(2) \begin{equation*} \hat{y} = \arg \min _{y^{\prime }} \underbrace{\sum _{i=1}^n (y_i^{\prime }{- y_i)^2}}_{\mbox{quality of fit}} + \lambda \underbrace{\sum _{i=1}^n \sum _{j=1}^n a_{ij}(y_i^{\prime } - {y_j^{\prime }})^2,}_{\mbox{smoothness}} \end{equation*}

in which we use λ to control the tradeoff between the two principles.

This expression can be compactly expressed using the Laplacian of *G*: *L* = *D* − *A* in which *D* is a diagonal matrix with node degree on the diagonal: *D*_*ii*_ = ∑_*j*_*a*_*ij*_. Then,

\begin{eqnarray*} f(\hat{y}) &=& \sum _{i=1}^n (\hat{y}_i-y_i)^2 + \lambda \sum _{i=1}^n \sum _{j=1}^n a_{ij}(\hat{y}_i - \hat{y}_j)^2\\ &=& ||\hat{y} - y||^2 + \lambda \hat{y}^TL\hat{y},\\ \frac{df}{d\hat{y}} &=& 2\hat{y} - 2y + 2\lambda L\hat{y} = 0,\\ \hat{y} &=& (I + \lambda L)^{-1} y. \end{eqnarray*}

The matrix (*I* + λ*L*)^−1^ is the *regularized Laplacian* of *G* (35). It is a positive semidefinite matrix and hence a kernel. In addition to the ‘guilt by association’ argument we note an additional reason from (27,36) that the regularized Laplacian is an appropriate tool for functional inference on protein interaction networks: it also naturally discounts paths that pass through high-degree nodes.

The combination of the multi-network kernel embedding described in the previous section with the Regularized Laplacian constitutes MUNK and the resulting cross-species similarity scores are MUNK*scores*. We denote the MUNK score of two proteins *p*_*i*_ and *p*_*j*_ as *d*_*ij*_, we refer to the RKHS in which *G*_1_ and *G*_2_ are embedded as MUNK-space, and the MUNK-representations are given by the rows of *C*_1_ and $\hat{C}_2$ (as defined in the previous section).

### Representations for protein (gene) pairs

We also find it useful to develop representations for pairs of nodes (proteins or genes). These can be used to capture functional similarity between two *pairs* of nodes across species. Further, pair-representations can then be used to predict outcomes for pairs of genes (e.g. synthetic lethality). Given two pairs of nodes (*v*_*i*_, *v*_*j*_), (*v*_*k*_, *v*_ℓ_), we define a pairwise similarity metric such that the score is large only if *d*_*ik*_ and *d*_*j*ℓ_ (or *d*_*i*ℓ_ and *d*_*jk*_) are both large. This reflects the hypothesis that synthetic lethal interactions occur within pathways, and between pathways that perform the same/similar essential biological function (37,38).

Hence, to represent a pair, we simply sum the MUNK-representations for the nodes in the pair. We then compare pairs by computing MUNK scores in the usual way. Given a matrix *C* of MUNK-representations for nodes, we define the MUNK-representations for a *pair* of nodes (*v*_*i*_, *v*_*j*_) as *P*_*C*_(*v*_*i*_, *v*_*j*_) = *c*_*i*_ + *c*_*j*_. Computing similarity for a two pairs (*v*_*i*_, *v*_*j*_) and (*v*_*k*_, *v*_ℓ_) then yields:

\begin{equation*} (c_i+c_j)^T(c_k+c_\ell ) = c_i^Tc_k+c_i^Tc_\ell +c_j^Tc_k+c_j^Tc_\ell . \end{equation*}

In general we expect each of the terms on the right hand side to be close to zero *unless* there is functional similarity between the corresponding nodes, because in high dimension, independent random vectors tend to be nearly orthogonal. We note that the pair-similarity scores and the pair-representations themselves can be used for a variety of tasks.

The utility of this approach is informed by recent work on predicting synthetic lethal interactions from network diffusion (20).

### Comparing to existing methods

We benchmark against state-of-the-art methods developed for network alignment and multi-species synthetic lethal classification. For network alignment we compare MUNK to two standard network alignment algorithms IsoRank and HubAlign (12,17). IsoRank was the first to combine local topology and sequence for the global network alignment problem (17). HubAlign identifies ‘topologically important’ genes and aligns these first before aligning the remaining genes (12). Both these methods serve as a natural comparison point for MUNK in that they use cross-species protein metrics that can be interpreted as similarity scores. We use default parameters for both IsoRank (using α = 0.6) and HubAlign (using α = 0.7 and λ = 0.1) using the authors’ publicly available software to produce cross-species matchings and similarity scores. We modified the HubAlign source code to output the similarity scores since it did not do so by default.

For multi-species synthetic lethal classification, we compare to SINaTRA (22). SINaTRA computes ‘connectivity profiles’ from a given network by computing graph theoretic measures of topology for each node, and then trains classifiers to predict synthetic lethal interactions across species from rank-normalized the connectivity profiles. We implemented SINaTRA in Python 3, as the authors did not make any software publicly available.

In order to demonstrate the advantages of the general-purpose representations constructed by MUNK we try to use all these existing algorithms for both cross-species functional similarity and predicting synthetic lethals. However, there is no obvious way to use the network alignment algorithms for multi-species synthetic lethal prediction. We do benchmark cross-species functional similarity using the connectivity profiles generated by SINaTRA.

### Data

For the experiments in this study, we study the human (*Homo sapiens*, or *H.s*.), mouse (*Mus musculus*, or *M.m*.), baker’s yeast (*S. cerevisiae*, or *S.c*.) and fission yeast (*S. pombe*, or *S.p*.) PPI networks.

#### Protein-protein interaction networks

We downloaded and processed PPI networks for human and mouse from the STRING database (39) PPI networks for *S.c*. and *S.p*. from BioGRID database (40) (refer to Supplemental Information S2.1 for details). We restricted each network to the two-core of the largest connected component and report summary statistics of the networks in Supplementary Table S1. We use the two-core of the graph because topologically indistinguishable nodes (nodes that participate in an automorphism of the graph) will necessarily have identical MUNK homology scores. A large class of topologically indistinguishable nodes includes many of the leaf nodes in the graph (degree-1 nodes). That is, in any case where there are two or more leaf nodes attached to the same parent, the nodes are topologically indistinguishable. Protein names were standardized by mapping to UniProt Accession IDs using mappings provided by the UniProt consortium (2).

#### Sequence homologs

For each combination of two organisms we identify homologous pairs of genes using NCBI’s Homologene database (10).

#### Protein function annotations

Protein functions were determined using the Gene Ontology database (GO) (1) using the annotations contained in the gene-to-term files. Currently GO contains >40,000 biological concepts over three domains: Molecular Function (MF), Biological Process (BP) and Cellular Component (CC). We use GO annotation corpora downloaded from SGD (41) for yeast and UniProt (2) for all other species. We exclude annotations based on IEA or IPI evidence due to their lower associated confidence levels. The gene-to-term mapping file from GO does not always include the more general terms implied by a specific gene-term pair (i.e. the ancestors of the term). Thus following (42) and unless otherwise noted we post-process obtained GO annotations by propagating and adding missing GO labels over ‘has part’ ‘part of’ and ‘is a’ GO term relations.

#### Synthetic lethal interactions

We constructed datasets of synthetic lethal interactions (SLI) and non-interactions (non-SLI) from two high-throughput studies of analogous proteins in baker’s (*S.c*.) and fission (*S.p*.) yeast (43,7). Also, following Jacunski *et al.* (22) we constructed datasets of SLI from BioGRID (v3.4.157) in *S.c*. and *S.p*. sampling an equivalent number of non-SLI from pairs in the PPI network without an SLI. We report the size of the datasets in Supplementary Table S2, and additional details in the Supplemental Information S2.2.

### Implementation

We implemented MUNK in Python 3 using the open-source NetworkX, NumPy and SciPy libraries (44,45). We executed software pipelines for performing the experiments in part using the Snakemake software (46). The source code for MUNK and experiments is available at https://github.com/lrgr/munk.

MUNK runs in a practical amount of time for networks of various sizes. Using an Intel Xeon E6-2660 v2 processor with 20 hyper-threaded cores (40 threads) and 94GB of memory MUNK-representations from human to mouse, mouse to human, human to *S.c*., *S.c*. to *S.p*. and *S.p*. to *S.c*., were computed in 249.1, 3.5, 5.4, 4.1 and 0.25 min, respectively.

## RESULTS

We demonstrate that MUNK-representations encode functional relationships between proteins in different species by performing three tasks: cross-species protein function annotation, multi-species synthetic lethal classification, and phenolog discovery. We also investigate procedures for choosing the homolog pairs that serve as landmarks, which we describe in *Landmark selection* below. Based on these investigations, we use the same set of 400 homolog-pairs at random to serve as landmarks in all other experiments.

### MUNK-representations capture functional similarity across species

Our results show that the similarity scores given by MUNK-representations are strongly correlated with functional similarity between human and mouse proteins. For this section we evaluate results for pairs of proteins (*p*_*i*_, *p*_*j*_), where *p*_*i*_ and *p*_*j*_ are from human (source) and mouse (target), respectively. We only include pairs for which neither *p*_*i*_ or *p*_*j*_ are part of a landmark pair.

We use the Resnik score (47) as a quantitative measure of functional similarity. The Resnik score between two Gene Ontology (GO) (1) terms is the information content of their most informative common ancestor in the GO hierarchy; to compare two proteins we take the maximum Resnik score over all pairs of GO terms. The Resnik score has been shown to be one of the best performing metrics for capturing functional similarity within the GO hierarchy (48).

**Figure 2.**
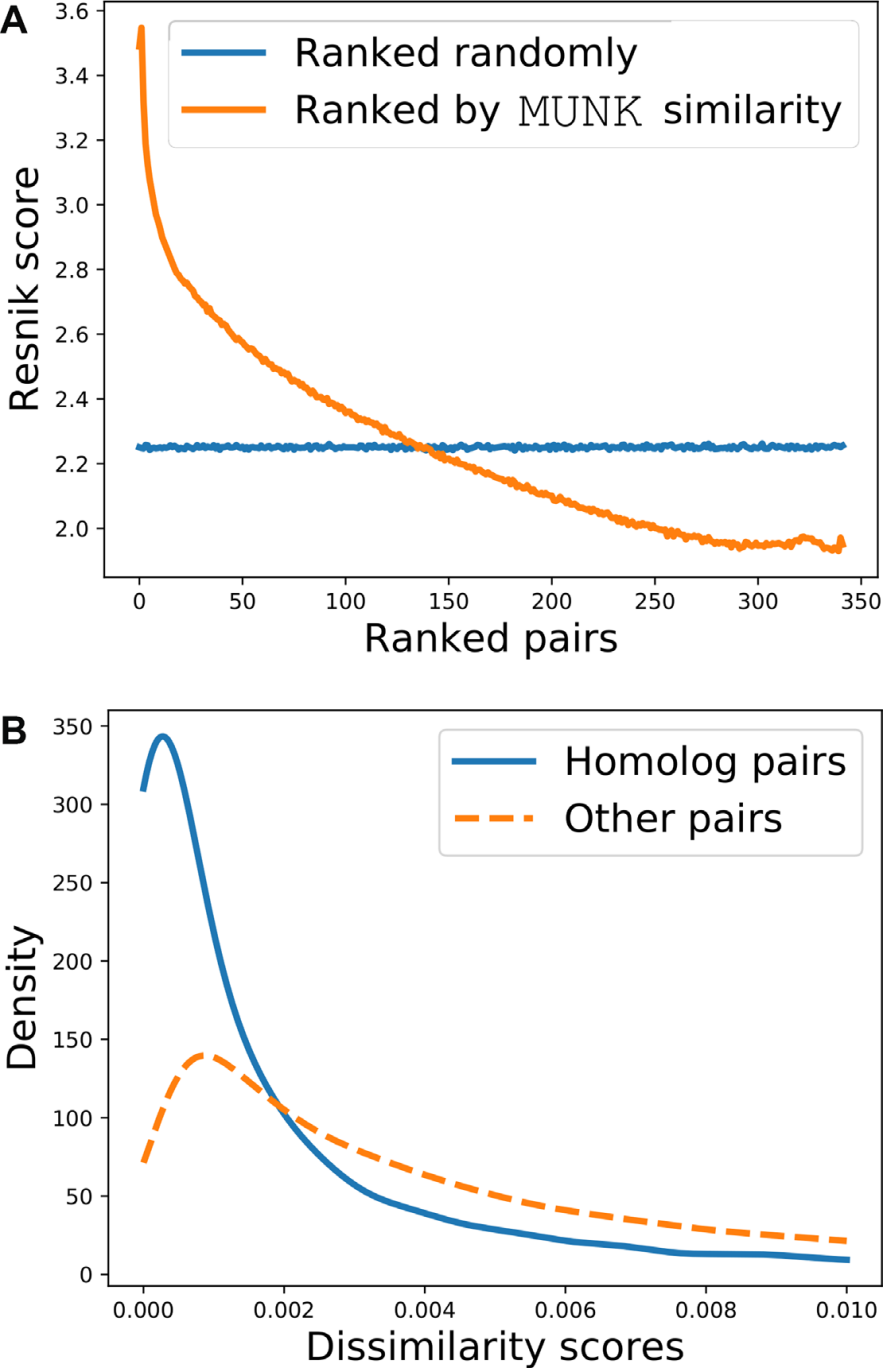
The relationship between MUNK similarity scores and functional similarity for the human (source) to mouse (target) embeddings. (**A**) Relationship between functional similarity measured by Resnik score (y-axis) and protein pairs ranked (x-axis) by MUNK similarity (shown in orange) and ranked randomly (shown in blue; included as a baseline). (**B**) Distribution of MUNK dissimilarity scores for homologous protein pairs compared to distribution for other (non-homologous) protein pairs.

To demonstrate the relation between MUNK similarity scores and functional similarity we order each pair according to their MUNK scores, and plot rankings against the Resnik score of the pair. The results (smoothed over non-overlapping windows of 100,000 observations) are shown in Figure 2A. Other cases are shown in Supplementary Information 3.1.

The figure shows that MUNK scores are strongly correlated with functional similarity across the entire range of scores. Furthermore, the very largest MUNK scores are indicative of protein pairs with particularly high functional similarity.

#### Homolog pairs have distinct MUNK similarity scores

Next, we show that pairs that are known to be functionally related are distinguishable by their MUNK similarity scores. For this purpose, we separate pairs (*p*_*i*_, *p*_*j*_) where *p*_*i*_ and *p*_*j*_ are homologous proteins in different organisms from other pairs.

In Figure 2B, we show the distribution of MUNK dissimilarity among known homolog pairs, as compared to the distribution of scores across other pairs. In this figure, we use reciprocal scores (dissimilarities), meaning that small scores are associated with high functional similarity. Only the left side of the distributions are shown, as the distribution of all pairs extends far to the right and obscures the homolog distribution on the left. As suggested by the plots, the mean MUNK dissimilarity scores for human-mouse homologs are 36% lower than the mean across other protein pairs. Other cases are shown in Supplementary Information S3.1. These results provide additional evidence that MUNK scores are correlated with functional similarity across species.

#### MUNK captures shared biological information beyond node degree

We find that MUNK captures shared biological information from network topology and not only from node degrees. We assess the statistical significance of the difference in MUNK similarity scores between homologous and non-homologous pairs by generating 1000 pairs of random networks in which each node is given degree very close to that in the original network, but in which edges have been randomized. Specifically, we follow the method of Newman *et al.* (49) to generate graphs with given degree distributions and remove self loops and parallel edges afterwards. We then compute an empirical *P*-value by counting the number of pairs of random networks for which the difference in mean MUNK similarity scores between homologs and non-homologs is greater than that observed in real PPI networks. Given the expense of generating many permutations of the large human network we instead assess two yeast (*S.c*. and *S.p*.) networks. We find that homologous pairs have statistically significantly higher MUNK similarity scores (*P* = 0.002 for embedding *S.c*. to *S.p*., and *P* = 0.005 for embedding *S.p*. to *S.c*.). Consequently, we conclude that the differences in the distributions of MUNK similarity scores for homologs and non-homologs is unlikely to be due to node degree alone, but instead is a result of MUNK capturing more detailed network topology.

#### Comparing MUNK to other methods

We compare MUNK to the IsoRank (17) and HubAlign (12) network alignment algorithms in their ability to predict cross-species functional similarity of proteins. We focus on two different measures of functional similarity. First is the Gene Ontology consistency (GOC) measure which is commonly used to evaluate functional network alignments produced by a matching algorithm. GOC measures the Jaccard index of the GO terms assigned to each pair of *matched* proteins (*p*_*i*_*p*_*j*_). For MUNK and IsoRank, we generate a matching of all proteins in the smaller network to one protein in the larger network using the Hungarian algorithm applied to the similarity scores. HubAlign automatically produces such a matching. Unlike MUNK, the protein similarity scores used by IsoRank and HubAlign are a convex combination of BLAST sequence similarity and network topological similarity between each cross-species pair of proteins. The main contribution of the algorithms we study here is in defining the cross-species network similarity, so it is crucial when evaluating alignment methods to control the relative amount of sequence versus network information used by the various methods. Accordingly, we compare the results for MUNK when using 400 cross-species landmark pairs with those of IsoRank and HubAlign when using sequence similarity scores only between the same 400 landmarks. However, for context, we also report results for IsoRank and HubAlign using all sequence similarity scores.

The second measure we use is *k*-functional similarity, which evaluates the *space* induced by the protein similarities and was used (without naming it) in (22). We define a pair of proteins (*p*_*i*_*p*_*j*_) from two species to be *k*-functionally similar if both *p*_*i*_ and *p*_*j*_ are annotated by the same GO term and, in each species, that GO term is associated with at most *k* proteins (see Data for details on processing of GO). We then rank cross-species protein pairs by similarity scores obtained from MUNK and other benchmarked algorithms and compute enrichment of *k*-functional similar pairs at *k* = 100 in the top ranked sets. We evaluate enrichment using area under the precision–recall curve (AUPR), which is an appropriate measure when there is a large class imbalance in the data (50) as is the case here (e.g. 3.9% out of 36,877,467 human-mouse protein pairs are classified as *k*-functionally similar at *k* = 100). We include comparisons to SINaTRA (22) and report the functional consistency (FC) of the computed matchings—an additional measure for evaluating network alignments (51,12)—in Supplementary Tables S4 and S5.

Table 1 shows functional measures for each algorithm for pairs of proteins from human-mouse human-baker’s yeast, and baker’s-fission yeast. When using all sequence similarity scores, IsoRank outperforms the other methods in terms of GO consistency (GOC) for human–mouse (0.515 for IsoRank versus 0.262 and 0.178 HubAlign and MUNK, respectively) and baker’s-fission yeast (0.288 for IsoRank versus 0.238 and 0.275 for HubAlign and MUNK, respectively), while HubAlign performs the best for human-baker’s yeast (0.223 for HubAlign versus 0.112 and 0.156 for IsoRank and MUNK, respectively). This may be due to IsoRank relying more on sequence similarity than the other methods, particularly for human-mouse, where 86% (3,621/4,217) of proteins in the mouse network have a homolog in the human network. When the algorithms are compared using the same amount of sequence information, MUNK outperforms HubAlign and IsoRank for human-baker’s yeast (0.156 for MUNK versus 0.119 for HubAlign) and baker’s-fission yeast (0.275 for MUNK versus 0.266 and 0.193 for IsoRank and HubAlign, respectively), and IsoRank outperforms for human-mouse (0.200 for IsoRank versus 0.178 and 0.115 for MUNK and HubAlign, respectively). Note that the IsoRank software did not produce a matching in human-baker’s yeast with restricted sequence information.

**Table 1.** Results for functional similarity measures of MUNK and network alignment algorithms HubAlign and IsoRank

Species	Algorithm	GOC	*k*-FS AUPR
human→ mouse	MUNK	0.178	0.060
	Iso Rank	**0.200** (0.515)	**0.075** (0.060)
	Hub Align	0.115 (0.262)	0.060 (0.067)
human→ baker’s yeast	MUNK	**0.156**	**0.034**
	Iso Rank	— (0.112)	— (0.022)
	Hub Align	0.119 (0.223)	0.025 (0.030)
baker’s → fission yeast	MUNK	**0.275**	0.067
	Iso Rank	0.266 (0.288)	**0.070** (0.045)
	Hub Align	0.193 (0.238)	0.046 (0.054)

GO consistency results are reported first restricting the algorithms to only use the same amount of sequence information (BLAST scores for the 400 ‘landmark’ homolog pairs used by MUNK), and then using all available sequence information for HubAlign and IsoRank. The reported results for MUNK are for embedding the smaller of the two networks into the larger one. IsoRank did not produce a matching in human-baker’s yeast with restricted sequence information.

Using *k*-functional similarity, we find that MUNK-space captures functional similarity comparably to HubAlign and IsoRank in different pairs of species (Table 1). For human-mouse, MUNK performs comparably to HubAlign but is outperformed by IsoRank (0.060 for MUNK versus 0.060 and 0.075 for HubAlign and IsoRank, respectively). In human-baker’s yeast, MUNK outperforms HubAlign by 26% (0.034 for MUNK versus 0.025 for HubAlign). In baker’s-fission yeast, MUNK performs comparably to IsoRank (achieving within 4% of IsoRank’s AUPR) and outperforms HubAlign by a large margin (0.067 for MUNK versus 0.070 and 0.046 for IsoRank and HubAlign, respectively). Thus, MUNK compares favorably to standard approaches for computing cross-species gene/protein similarity, but has a key additional advantage of creating general-purpose protein representations.

As a baseline, we use BLAST bit-scores between protein sequences as similarity scores and evaluate *k*-functional similarity using these scores. MUNK and IsoRank perform comparably to BLAST which achieved AUPR of 0.064, 0.029, 0.062 for human-mouse, human-baker’s yeast and baker’s-fission yeast, respectively.

To investigate novel functional matches between cross-species pairs with low sequence similarity, we repeated the GO consistency analysis for only non-homologous pairs. Supplementary Table S3 shows the results. Each method identified different fractions of these potential novel matches, as each method identified different numbers of homolog pairs. As expected, the average GOC scores dropped for each method, but the relative ranking of the methods remained consistent with only one change; IsoRank performed better than MUNK for baker’s-fission yeast.

#### Landmark selection

We investigate two ways of choosing landmark pairs of homologs for each pair of species we analyzed. Motivated by the idea that hubs may serve as good landmarks as they are close to most nodes, we select landmark pairs in order of their average rank by degree in each respective species. We also select landmark pairs completely at random. We compare the two approaches while varying the number of landmarks by computing the average GO consistency (GOC) score of the MUNK-space given by the set of landmarks. For each number of landmarks, we sample ten random sets. We note that we are limited in the number of landmarks we can use to the number of homologs shared by the pair of species, and restrict our experiments to using up to 1000 landmarks. We further note that homologous nodes tend to have higher degree than non-homologous nodes in each species (Supplementary Figure S3).

Supplementary Figure S4 shows that there is little difference between the two selection procedures. Selecting landmarks by degree only seems to provide an advantage when the number of landmarks is small (≤ 100), possibly indicating that choosing >100 nodes at random means most nodes are close to at least one landmark. We also find that GOC performance plateaus in human-mouse, human-baker’s yeast, and baker’s-fission yeast species when we include 1000, 200 and 300 landmarks, respectively. Interestingly, this does not indicate that larger networks require more landmarks as, out of the three pairs of species tested, human-baker’s yeast has the most combined number nodes (18,481) but plateaus with the fewest landmarks. However, the human-mouse network did not have an obvious plateau, this is perhaps because a very large fraction of mouse nodes have a human homolog (86%). In all our remaining experiments, we use 400 random landmarks, as in each pair of species MUNK using 400 random landmarks achieved a GOC score within 0.02 of the maximum.

#### Leveraging multiple model organisms for function prediction

We also study the potential for leveraging annotations from multiple model organisms simultaneously using MUNK-representations. This problem has not previously been explored extensively, with the exception of (52) which took a Bayesian approach. Our intent is not to propose a new method for function prediction, but to further demonstrate the value of cross-species information as obtained via MUNK.

We focus on function prediction in three species: human, mouse, and baker’s yeast (*S.c*.). Following the approach taken in (26) we focus on predicting rare GO terms—in particular we form predictions for all GO terms that occur between 2 and 300 times in the annotation corpus of one species, using the specific annotations from the GO gene-to-term mapping files. We study the information content of multiple MUNK scores by constructing a convex combination α_*H*_, α_*Y*_, α_*M*_ of a given species’ diffusion score *D*_1_ with the MUNK similarity scores *D*_12_ of the two other species.

We perform a binary classification for each GO term. We rank proteins by the total scores contributed by other proteins—within the same and across species—annotated with that term, weighted by α_*H*_, α_*Y*_, α_*M*_. We then assess performance using the maximum *F*_1_ score averaged over all GO terms. We test whether such a convex combination has greater predictive power for functional inference than just using information from any single species. Details of our method are given in Supplementary Information S1.2.

**Figure 3.**
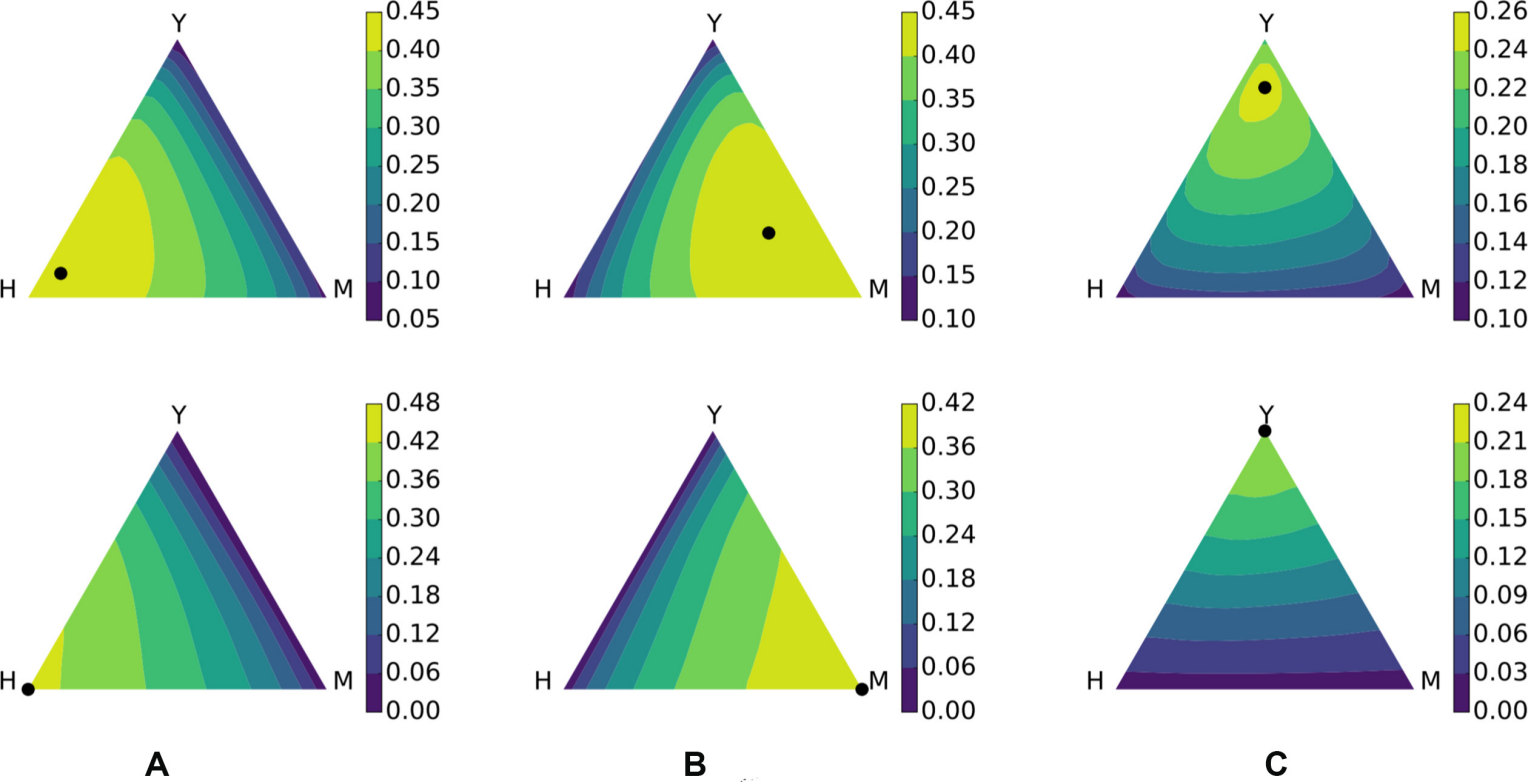
*F*_1_ score of functional inference using multiple additional species. Inferring protein functions for (**A**) human—H, (**B**) mouse—M and (**C**) baker’s yeast—Y. Upper row: using MUNK scores; Lower row: null hypothesis, using randomly permuted MUNK scores.

Figure 3 shows the prediction performance obtained over the simplex (α_*H*_, α_*Y*_, α_*M*_) such that ∑α_*i*_ = 1. Contours show *F*_1_ scores, and the point of maximum *F*_1_ score is plotted. For comparison purposes we provide results for the case in which MUNK scores are randomly permuted. The figure shows that in each case, the greatest improvement in functional prediction comes when making use of information from both additional species. The improvements in functional prediction are shown in Supplementary Table S8.

### Multi-species synthetic lethal classification with MUNK-representations

In this section we demonstrate the advantages of general-purpose cross-species protein representations by using MUNK-representations to predict synthetic lethal interactions (SLI) in multiple species simultaneously. Existing matching-based network alignment methods are unable to generalize to this problem, since SLI are a property of *pairs* of genes. Similarly, most existing methods for creating network-based representations are also ill-suited for this problem, since genes in different species are in different vector spaces with different dimensions. The exception to this is the SINaTRA algorithm (22) which we benchmark against.

We show that classifiers trained on MUNK-representations can accurately predict SLI in two different species of yeast—*S. cerevisiae* (*S.c*.) and *S. pombe* (*S.p*.)—simultaneously, providing evidence that gene pairs with SLI in different species are co-located in MUNK-space. More specifically, we train a random forest (RF) to classify gene pairs as SLI or non-SLI within both species *simultaneously*, using the source embedding (given by $P_{C_1}$) and the target embedded into source space (given by $P_{\hat{C}_2}$); see the discussion under *Representations for protein (gene) pairs* for details. We perform 4-fold cross-validation, fixing the relative fraction of pairs from each species, and assess the degree of separation between SLI and non-SLI in MUNK-space by evaluating the RF classifications with maximum *F*_1_ score (the harmonic mean of precision and recall), the area under the ROC curve (AUROC), and the area under the precision-recall curve (AUPR). We report the average across the four folds, separating the results by species. We use a nested cross-validation strategy to choose the number of trees for the RF that maximizes the held-out AUPR. For simplicity, all of our experiments in this section use *S.c*. as the source and *S.p*. as the target.

We first train classifiers on an SLI dataset of high-confidence, low-throughput interactions from BioGRID (40) (see *Data* for additional details of the dataset). This dataset is the most recent update of the dataset used by Jacunski et al. (22) and we follow their approach by creating a dataset with an equal number of non-SLI sampled randomly from pairs in the PPI networks without SLI. Table 2 shows the results, which demonstrate that classifiers trained on MUNK-representations are very accurate at discriminating between SLI and non-SLI. The RFs achieve average AUROC of 0.933 in *S.c*. (0.876 in *S.p*.), AUPR of 0.933 (0.877), and maximum *F*_1_ score of 0.860 (0.814). We then compare the results using MUNK features to SINaTRA (which also uses a RF), using the same protocol as above but train RFs with the rank-normalized features produced by SINaTRA for cross-species predictions from (22). SINaTRA achieves average AUROC of 0.908 in *S.c*. (0.880 in *S.p*.) AUPR of 0.907 (0.892), and maximum *F*_1_ score of 0.834 (0.808). Thus, while both sets of classifiers make highly accurate predictions, the RFs trained using MUNK-representations as features outperform SINaTRA on four of six measures.

**Table 2.** Results training classifiers for synthetic lethal interactions on baker’s yeast (*S.c*.) and fission yeast (*S.p*.) data *simultaneously*

Dataset	Test	Features	AUROC	AUPR	Max *F*_1_
BioGRID (40)	*S.c*.	MUNK	**0.933**	**0.933**	**0.860**
		SINaTRA	0.908	0.907	0.834
	*S.p*.	MUNK	0.876	0.877	**0.814**
		SINaTRA	**0.880**	**0.892**	0.808
Chromosome biology (7,43)	*S.c*.	MUNK	**0.864**	**0.402**	**0.421**
		SINaTRA	0.788	0.201	0.282
	*S.p*.	MUNK	0.822	0.370	0.423
		SINaTRA	**0.837**	**0.393**	**0.437**

We compute performance separately for each species (indicated by ‘Test species’). For each statistic, we report the average on held-out data from 4-fold cross-validation, and bold the highest (best) score.

Next we train linear support vector machines (SVMs) instead of RFs to learn hyperplanes separating SLI and non-SLI in MUNK-space, and only see a small degradation in performance compared to the RF (Supplementary Table S6). This further supports the case that SLI in different species are co-located in MUNK-space because, unlike the decision boundary learned by the RF, the SVM learns a linear decision boundary. Therefore, because the linear SVM also classifies SLI with high accuracy in both species simultaneously, there is evidence for a direction in MUNK-space capturing synthetic lethality.

We then train random forest classifiers with matched high-throughput datasets from *S.c*. and *S.p*. These datasets consist of SLI and non-SLI pairs among 743 *S.c*. genes (43) and 550 *S.p*. genes (7) involved in chromosome biology (see *Data* for additional details of the dataset). The key differences between the chromosome biology SLI datasets and the BioGRID datasets are that the chromosome biology datasets are restricted to functionally similar genes include 5.5% SLI and 94.5% measured non-SLI in *S.c*. and 10.6% SLI and 89.4% measured non-SLI in *S.p*. (unlike the BioGRID data which only measured SLs), and were generated through high-throughput experiments.

Table 2 shows that the RFs trained on MUNK-representations achieve significant predictive performance on held-out data from the chromosome biology dataset, with an AUROC of 0.864 in *S.c*. (0.822 in *S.p*.), AUPR of 0.402 (0.370), and maximum *F*_1_ score of 0.421 (0.423). While these results show significant predictive power, the performance of the classifiers is poorer than on the BioGRID data. This is likely due to a combination of the noisy, high-throughput nature of the data measurements and the class imbalance. Interestingly, on the chromosome biology dataset, MUNK outperforms SINaTRA by a large margin for predictions in *S.c*., while SINaTRA outperforms MUNK by a smaller margin for predictions in *S.p*. Finally, we repeat our analyses to evaluate how well our classifiers generalize to held-out genes on both datasets (as suggested by (53)), and while there is a drop in performance, they retain significant predictive power (see Supplementary Information S3.2 and Supplementary Table S7).

Together, these results show that synthetic lethal interactions are significantly clustered *across species* in MUNK-space, and that by using MUNK-representations, which leverage knowledge of a subset of homologous genes across species, classifiers can make accurate predictions for other homologous and non-homologous gene pairs.

### Using MUNK-homologs to find known and novel phenologs

As a third demonstration of the utility of general-purpose protein representations, we use MUNK to develop a broadened notion of *phenolog*, generalizing the definition in (5). As put forward in (5) a phenolog is a pair of homologous phenotypes in different species whose identification can yield, e.g. non-obvious disease models. That paper operationally defined a phenotype based on over-representation of homologous proteins associated with each phenotype. Here, we argue that over-representation of *functionally-similar* proteins constitutes a more powerful operational definition for a phenolog.

Because MUNK scores are strongly correlated with functional similarity across a range of scales (Figure 2A), MUNK scores generalize the relationship of two proteins in different species from a binary value (homologous or not) to a continuous value (degree of functional similarity). This is useful because, while reliance on homologs is a good start for determining phenotypic preservation, the requirement of sequence preservation may be too restrictive if the primary goal is to study function across species (54–56). Accordingly, in comparing two phenotypes in different species, we can ask about over-representation of functionally-similar proteins over a wide range of functional similarity. We hypothesize that examining different degrees of functional similarity can expose different kinds of phenologs.

We investigate whether additional phenologs may be discovered through the expanded notion of homology that is provided by MUNK (rather than reproduce the results in (5) with a different methodology). As a proof of concept we show results using human-mouse MUNK scores. To make comparisons with (5) we use the same phenotype to gene association datasets as in that study.

We threshold MUNK similarity scores leading to a binary classification of each cross-species gene pair as to whether it is functionally-similar at the chosen threshold level. In the results we report here, we set the threshold so as to output a small set of phenolog predictions. As discussed, other threshold settings would potentially produce different functional classes of phenologs as the pairs of genes considered close changes. To compare a phenotype in one species to a phenotype in a different species we count the number of functionally-similar pairs across species in the two associated phenotypes. We then use the same procedure as in (5) with functionally-similar pairs playing the role that homologs did in that work; details are provided in Supplementary Information S1.3.

By using a stringent threshold on MUNK scores, we sharply limit the size of the set of phenologs predicted. Whereas (5) reported 3634 phenotype pairs passing significance testing our results show 47 pairs of phenotypes to be significantly associated. Within this set were 18 phenologs previously reported by (5) which is not surprising given that many homolog pairs are ranked highly by MUNK (e.g. see Figure 2b). However our primary interest are the matches that are not part of the homolog-based phenologs reported in (5).

**Figure 4.**
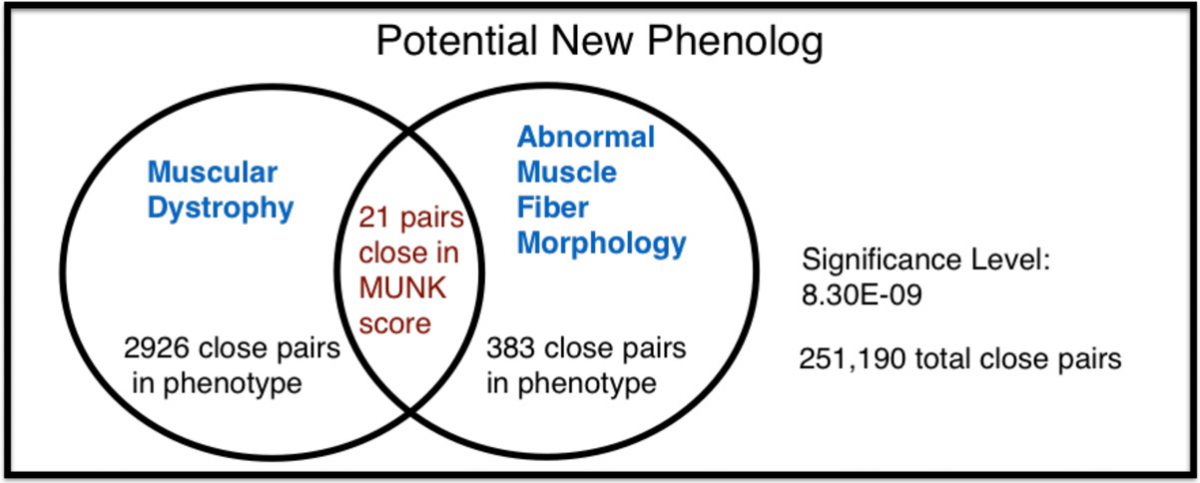
A potential new phenolog, not found by (5) relating the phenotypes Muscular Dystrophy (human) and Abnormal Muscle Fiber Morphology (mouse).

We find that MUNK-based similarity can uncover many new phenologs that are not statistically significant when using homologs. As an example we show in Figure 4 details of a phenolog found using MUNK, but not found in (5). This phenolog matches *Abnormal Muscle Fiber Morphology* in the mouse with *Muscular Dystrophy* in human. The example is illustrative as it shows that false negatives can occur when using only homologs even for straightforward matches such as this one. In fact the homolog-based method used in (5) finds only 1 homolog in common while MUNK detects 21 functionally-similar pairs of proteins. The larger set of functionally-similar protein pairs uncovered by MUNK gives greater statistical power for cases such as this one, where there is only a small number of homologs shared between the two phenotypes.

We find that many of the remaining 29 phenotype pairs identified using MUNK are potentially valid phenologs. To demonstrate, we compare the human phenotype (disease) with mouse phenotype (symptom). The 29 pairs of phenotypes ranged over 8 unique human diseases and 24 unique mouse phenotypes. We obtained the description of each disease from the Genetics Home Reference [https://ghr.nlm.nih.gov/] and compared the disease description to the matched mouse phenotypes.

Table 3 groups the results into three categories: obvious symptom match, possible symptom match, and novel match. For each disease term and mouse phenotype pair, if the name of the mouse phenotype was indicated as a symptom in the disease description, we considered an obvious match; if the disease description contained a symptom that was similar to the mouse phenotype, we considered it a possible match; and if there was no similarity in the phenotype and the disease description we considered it as a novel match. Table 3 shows that, for over 75% of the new phenologs predicted using MUNK, there is evidence in the literature supporting the association.

**Table 3.** Matched mouse phenotypes per human disease among newly-identified phenologs

Human disease	Obvious Match	Similar Match	Novel Match
Systemic lupus erythematosus	1	0	0
Dilated cardiomyopathy	4	0	1
Zellweger syndrome	2	1	2
Dysfibrinogenemia	0	0	1
Myopathy	1	0	0
Bardet-Biedel syndrome	6	2	1
Adrenoleukodystrophy	0	3	1
Muscular Dystrophy	2	0	1
**Total**	**16**	**6**	**7**

In summary, we find that functionally-similar pairs as identified via MUNK can indeed provide a basis for expanding the set of phenologs discovered by previous methods. We anticipate future research developing methods to uncover phenologs may use previous methods in tandem with MUNK-based methods to more thoroughly explore the space of possible phenologs.

## DISCUSSION AND FUTURE WORK

We introduce a novel, kernel-based algorithm to create general-purpose, multi-species protein representations using biological networks and sequence data. We use a particular diffusion kernel—the regularized Laplacian—to create functional representations, and use the resulting algorithm, MUNK, to embed proteins from humans, mice, and yeast into shared spaces. We evaluate the MUNK-representations on cross-species functional similarity and multi-species synthetic lethal prediction, showing the MUNK-representations lead to comparable performance as specialized methods for these tasks. We also use MUNK to expand the notion of orthologous phenotypes beyond evolutionarily conserved sequence and identify known and novel phenologs, providing evidence for non-obvious human disease models. Importantly, in these tasks, we transfer knowledge both from humans to model organisms and from model organisms to humans. Thus, MUNK represents a new direction towards realizing the crucial goal of algorithms for transferring knowledge of genetics across species.

The phenologs found using MUNK representations motivate a larger investigation into non-obvious cross-species phenotype relationships. While the seminal work of McGary *et al.* (5) was based on the hypothesis that conserved molecular functions can produce different ‘organism-level’ phenotypes our results show that phenotypes can have statistically significant relationships at a broader level of protein function. This may suggest that the functional relationships between phenotypes in different species exist on a continuum, resulting in different types of disease models.

Our approach of creating cross-species protein representations can be seen as a component of a *transfer learning* (57) approach for cross-species inference. The promise of transfer learning—using knowledge gained in solving one task to aid in solving a different task—for cross-species inference is to leverage species where data is widely available for predictions in species where data is sparse. For example, this is the case for genetic interactions, where ∼90% of pairwise genetic interactions have been measured in baker’s yeast (58) while fewer than 1% of pairs have been tested in humans. Transfer learning is often approached by finding appropriate transformations of data features (e.g. ‘domain adaptation’, e.g. see (59)). For MUNK methods for aligning the source and target embeddings may be required to make such a transfer learning approach possible. At the same time we showed that methods for transferring knowledge across species can be useful even when there is a wealth of data in the target species. Thus to achieve optimal performance, supervised learners may need to train on multiple species simultaneously.

Beyond kernels derived from protein interaction, there are a wide range of other kernels that can inform biological function assessment, including kernels derived from co-expression, genetic interaction, metabolic pathways, domain structure, and sequence (36,60,61). Because MUNK is a method for creating a new kernel encompassing the nodes of multiple networks, it holds potential as a new tool for kernel learning methods such as support vector machines in a wide variety of applications beyond cross-species function prediction.

## Supplementary Material

Supplementary Data
