# Supplementary material for: Functional protein representations from biological networks enable diverse cross-species inference
Source: Nucleic Acids Res. 2019 Mar 8;47(9):e51. doi: 10.1093/nar/gkz132 (PMC6511848; doi:10.1093/nar/gkz132)
Supplement: Supplementary Data [file gkz132_supplemental_file.pdf]

Published online 2019

Nucleic Acids Research, 2019
doi:10.1093/nar/gkz132

Supplementary Information: Functional Protein Representations from Biological Networks Enable Diverse Cross-Species Inference

Jason Fan [1], Anthony Cannistra [2], Inbar Fried [3], Tim Lim [4], Thomas Schaffner [5], Mark Crovella [4], Benjamin Hescott [6,†], and Mark D.M. Leiserson [1,†*]

[1]Department of Computer Science and Center for Bioinformatics and Computational Biology, University of Maryland, College Park, [2]Department of Biology, University of Washington, [3]University of North Carolina Medical School, [4]Department of Computer Science, Boston University, [5]Department of Computer Science, Princeton University, and [6]College of Computer and Information Science, Northeastern University
[†]Equal contribution

Received July 27, 2018; Revised January 09, 2019; Editorial Decision February 14, 2019; Accepted February 18, 2019

SUPPLEMENTARY INFORMATION

S1 Methods

S1.1 Parameter choice For all regularized Laplacians, we used a value of $\lambda = 0.05$. We found that the resulting relationship between Resnik similarity and MUNK similarity score did not vary significantly for λ values between 0.005 and 0.1.

S1.2 Function Prediction Methods We assess prediction accuracy using leave-one-out cross validation. Let $K^s = \{k^s_{ij}\}$ denote the regularized Laplacian for species s. For GO term g, let $\mathcal{G}^s_g$ be the set of proteins in species s that are annotated with g. The same-species annotation score for a given protein p and GO term g is:

$$c^s(p,g) = \frac{1}{|\mathcal{G}^s_g|} \sum_{i \in \mathcal{G}^s_g} k^s_{pi}$$

in which p is excluded from the sum (i.e., if it is contained in $\mathcal{G}^s_g$). We also construct a cross-species annotation score for each protein, in which MUNK scores with respect to proteins in the other organism are used:

$$c^{s_0,s_1}(p,g) = \frac{1}{|\mathcal{G}^{s_1}_g|} \sum_{i \in \mathcal{G}^{s_1}_g} d_{pi}$$

where $d_{pi} = D_{12}(p,i)$ is the MUNK score for protein p in species s_0 and protein i in species s_1. The prediction score is then $h(p,g) = \alpha c^{s_0}(p,g) + (1-\alpha) c^{s_0,s_1}(p,g)$. To use multiple cross-species annotations, say n, we generalize $h(p,g)$ to a convex combination of the same- and cross-species annotation scores: $h(p,g) = \alpha_0 c^{s_0}(p,g) + \sum_{i=1}^n \alpha_i c^{s_0,s_i}(p,g)$ such that $\sum_{i=0}^n \alpha_i = 1$.

We evaluate predictions using area under the receiver operating curve (AUC) and maximal F-score (over all detection thresholds). Since we are concerned with predicting rare GO terms, we find that maximal F-score is generally a more discriminative metric. We set the convex coefficients $\{\alpha_i\}$ via cross-validation.

S1.3 Phenolog Discovery Our method matches that used in (1), using protein pairs with high MUNK similarity scores rather than homologs obtained from Homologene. (Note that none of the landmarks (which are a subset of the homologs) are used to discover new phenologs.) Specifically, let P_1 be the genes associated with the phenotype in species 1 and P_2 be the genes associated with the phenotype in species 2. Our contingency table consists of the counts of the number of 'MUNK-homologs' involving $P_1 \cap P_2$, $P_1 \setminus P_2$, $P_2 \setminus P_1$, and $(\Omega \setminus P_1) \setminus P_2$, with Ω denoting the set of all close pairs. We used a Fisher exact test to measure significance, and considered the match significant if the uncorrected P-value was less than 0.05. We corrected for multiple testing using a Bonferroni correction; there were $1{,}278{,}312$ possible phenotype matches so we set the significance level at 3.9×10^{-8}.

S2 Data

S2.1 Protein-protein interaction networks We constructed protein-protein interaction (PPI) networks in *S.c.*, *S.p.*, mouse, and human. The *S.c.* and *S.p.* networks were obtained from the Biological General Repository for Interaction Datasets (BioGRID) (2) version 3.4.157. Mouse and human PPIs were obtained from the STRING database version 9.1 (3). PPI networks obtained were processed by mapping the protein names to the same namespace. Genes that could not be mapped via the UniProt database were removed from the PPI networks entirely. We provide further details of the network processing below. Table S1 shows summary statistics for the PPI networks before and after processing.

S2.2 Synthetic lethal interactions We constructed datasets of synthetic lethal interactions (SLI) in *S.c.* and *S.p.* from published epistatic miniarray profiles (E-MAPs). E-MAPs

*To whom correspondence should be addressed: mdml@cs.umd.edu

© 2019 The Author(s)

include genetic interactions scores for pairs of genes, where the magnitude of the score reflects the strength of the genetic interaction. We downloaded E-MAPs for *S.c.* from the supplementary information of Collins, et al. (4), and for *S.p.* from the supplementary information of Roguev, et al. (5). We classified each pair of genes in each E-MAP as SLI, non-SLI, or uncertain. We used the thresholds from the Collins, et al. (4) supplementary information to classify pairs in *S.c.*. Given a pair with E-MAP score ϵ, we classified it as SLI if $\epsilon < -3$, uncertain if $-3 \leq \epsilon < -1$, and non-SLI otherwise. Similarly, we used the threshold for synthetic lethality from the Roguev, et al (5) supplementary information and used the same threshold for uncertainty, classifying *S.p.* pairs as SLI if $\epsilon < -2.5$, uncertain if $-2.5 \leq \epsilon < -1$, and non-SLI otherwise. We also remove pairs of genes in which either gene is not found in the corresponding PPI networks described in the main text. The resulting datasets included 7,165 SLI and 123,507 non-SLI in *S.c.*, and 5,599 SLI and 97,541 non-SLI in *S.p.*

We also constructed datasets of SLI from BioGRID (2) (v3.4.157). For both *S.c.* and *S.p.*, we extracted interactions of type 'Synthetic Lethality' only (i.e. ignoring other negative genetic interactions), yielding datasets of 13,645 and 908 SLI, respectively.

We then standardized the datasets by mapping genes names to Uniprot Accession IDs (6). Genes that could not be mapped via UniProt were excluded for this study, as were those that were not found in the processed PPI networks. For the BioGRID SLI dataset, we followed Jacunski, et al. (7), by sampling an equivalent number of non-SLI pairs from genes PPI networks that do not partake in SLI in the BioGRID dataset. Table S2 shows summary statistics of the SLI datasets before and after processing.

S3 Results

S3.1 Associations of MUNK *scores with functional similarity for other pairs of species* We associated MUNK similarity scores and functional similarity for pairs of proteins in additional pairs of species, using the methodology described in the main text. Figures S1 and S2 show the results for embedding human into yeast, and mouse into yeast.

S3.2 Evaluating the generalization of synthetic lethal interaction classifiers to held-out genes Predicting synthetic lethal interactions between gene pairs using features constructed for individual genes is an example of a pair-input classification problem. A challenge with evaluating classifiers trained on pair inputs with held-out data is that, for a given pair (u,v), it is possible that the features for only u, only v, both u and v, or neither u and v, can be found in the training data (8). Thus, information concerning genes found in the held-out data may be 'leaked' to the classifier during training. To evaluate the effect of this issue, Park & Marcotte (8) suggest evaluating classifications for gene pairs which contain one, two, or no genes in the training data separately. This is analogous to holding out individual *genes* instead of *gene pairs* at training time and, thus, we evaluate the effect of pair-inputs by repeating the experiments above but hold out genes instead of gene pairs for evaluation. We report the results in Table S7. We find that the classifiers are able to predict SLI for genes *not* found in the training data, but with a significant change in performance compared to genes found in the training data. On the BioGRID dataset, the classifiers achieve an AUROC of 0.872 in *S.c.* (0.823 in *S.p.*), an AUPRC of 0.875 (0.814), and maximum F_1 of 0.797 (0.772). On the chromosome biology dataset, the classifiers achieve an AUROC of 0.701 in *S.c.* (0.691 in *S.p.*), an AUPRC of 0.160 (0.207), and maximum F_1 of 0.202 (0.285). We hypothesize that the larger drop in performance on the chromosome biology data is due to the matched nature of the *S.c.* and *S.p.* datasets. We also find similar drops in performance for SINATRA when holding out genes instead of pairs (also in Table S7).

S4 Supplementary Tables

Table S1. Summary statistics of PPI networks. We processed the graphs to restrict to the two-core of the largest connected component.

Species	Source	Processing	Nodes	Edges
Baker's yeast (*S.c.*)	BioGRID (2)	Before	5,961	99,539
		After	5,609	95,997
Fission yeast (*S.p.*)	BioGRID (2)	Before	2,888	9,433
		After	1,865	7,712
Human	STRING (3)	Before	15,129	155,866
		After	12,872	153,609
Mouse	STRING (3)	Before	6,596	18,697
		After	4,217	16,318

Table S2. Summary statistics of synthetic lethal interaction datasets.

Species	Reference	Processing	Total	SLs	Non-SLs	Uncertain
Baker's yeast (*S.c.*)	Collins, et al. (4)	Before	150,636	7,240	125,927	17,469
		After	129,385	7,112	122,273	0
	BioGRID v3.4.157 (2)	Before	16,630	16,630	0	0
		After	27,050	13,525	13,525	N/A
Fission yeast (*S.p.*)	Roguev, et al. (5)	Before	118,248	5,754	101,595	10,899
		After	24,214	2,556	21,658	0
	BioGRID v3.4.157 (2)	Before	1,020	1,020	0	0
		After	1,316	658	658	N/A

Table S3. GOC of aligned gene pairs computed from MUNK-similarity scores and network alignment algorithms HUBALIGN and ISORANK. The GOC scores reported are for non-homologs only (pairs with weak sequence similarity). Homolog pairs recovered from the alignments are removed from the analysis above. For these results, the algorithms are restricted to only use the same amount of sequence information (BLAST scores for the 400 'landmark' homolog pairs used by MUNK). The reported results for MUNK are for embedding the smaller of the two networks into the larger one. Note that the ISORANK software did not produce an alignment for human-baker's yeast with restricted sequence information.

Species	Algorithm	Non-homologs matched	GOC
human→ mouse	MUNK	3696	0.126
	ISORANK	3623	**0.145**
	HUBALIGN	4041	0.103
human→ *S.c.*	MUNK	5292	**0.135**
	ISORANK	−−−	−−−
	HUBALIGN	5294	0.098
S.c.→ *S.p.*	MUNK	1457	0.204
	ISORANK	1642	**0.229**
	HUBALIGN	1756	0.171

Table S4. Functional consistency (FC) of aligned gene pairs computed from MUNK similarity scores and network alignment algorithms ISORANK and HUBALIGN. For ISORANK and HUBALIGN, FC scores are computed for alignments computed using the same amount of sequence similarity information as used for MUNK (BLASTscores for only 400 landmark pairs). Following (9), FC scores are computed using the specific gene-to-term GO labels given in the Gene Ontology gene-to-term file.

Species	No. of shared GO terms	MUNK	ISORANK	HUBALIGN
human→ mouse	≥1	55.9	60.6	42.7
	≥2	31.3	37.4	19.1
	≥3	19.2	24.8	8.9
	≥4	14.2	18.8	5.1
	≥5	11.4	14.8	3.6
human→ *S.c.*	≥1	37.1	–	27.4
	≥2	15.8	–	9.9
	≥3	7.9	–	5.0
	≥4	4.3	–	2.9
	≥5	2.4	–	1.8
S.c.→ *S.p.*	≥1	53.8	53.3	38.0
	≥2	29.9	26.4	12.5
	≥3	17.5	13.9	5.7
	≥4	9.3	6.1	2.5
	≥5	4.0	3.0	0.8

Table S5. k-functional similarity results for SINATRAand BLAST. For SINATRA, we compute similarity scores using Euclidean distance following (7). For BLAST, we directly interpret bitscores as similarity scores.

Species	Algorithm	AUPR
human→ mouse	SINATRA	0.043
	BLAST	0.064
human→ *S.c.*	SINATRA	0.018
	BLAST	0.029
S.c.→ *S.p.*	SINATRA	0.041
	BLAST	0.062

Table S6. Results training linear support vector machines to classify synthetic lethal interactions on *S.c.* and *S.p.* data *simultaneously*. We compute performance separately for each species (indicated by 'Test species'). For each statistic, we report the average on held-out data from 4-fold cross-validation over *gene pairs*, and bold the highest (best) score.

Dataset	Test species	Algorithm	AUROC	AUPRC	Max F_1
BioGRID (2)	*S.c.*	MUNK	**0.953**	**0.933**	**0.892**
		SINATRA	0.798	0.774	0.750
	S.p.	MUNK	0.714	0.657	0.731
		SINATRA	**0.786**	**0.846**	**0.850**
Chromosome biology (4, 5)	*S.c.*	MUNK	**0.865**	**0.334**	**0.396**
		SINATRA	0.631	0.092	0.156
	S.p.	MUNK	**0.711**	0.123	**0.208**
		SINATRA	0.569	**0.124**	0.214

Table S7. Results training random forests to classify synthetic lethal interactions on *S.c.* and *S.p.* data *simultaneously*. We compute performance separately for each species (indicated by 'Test species'). For each statistic, we report the average on held-out data from 4-fold cross-validation over *genes*, and bold the highest (best) score.

Dataset	Test species	Algorithm	AUROC	AUPRC	Max F_1
BioGRID (2)	*S.c.*	MUNK	**0.872**	**0.875**	**0.797**
		SINATRA	0.848	0.852	0.779
	S.p.	MUNK	0.823	0.814	**0.772**
		SINATRA	**0.839**	**0.855**	0.769
Chromosome biology (4, 5)	*S.c.*	MUNK	**0.701**	**0.160**	**0.202**
		SINATRA	0.681	0.115	0.184
	S.p.	MUNK	0.691	0.207	0.285
		SINATRA	**0.723**	**0.239**	**0.314**

Table S8. Improvement in functional prediction using two other species.

**Target Species**	AUC	F_1 score
Human	0.3%	2.6%
Mouse	1.0%	8.6%
Baker's yeast	0.3%	16.0%

S5 Supplementary Figures

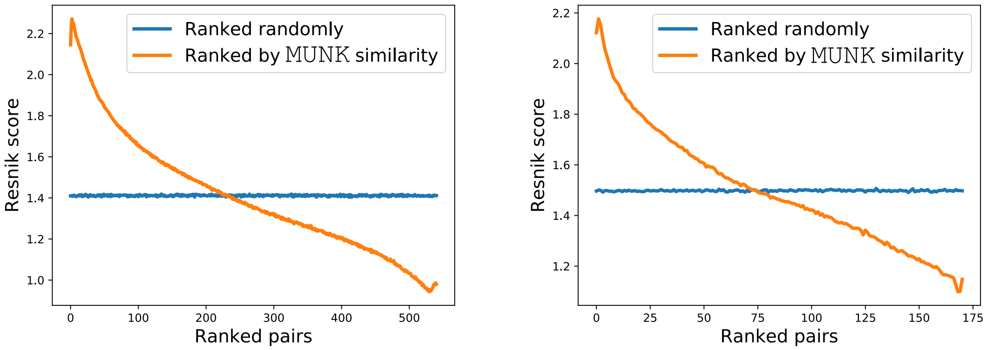

(a) (b)

Figure S1. Relationship between cross-species Resnik similarity and MUNK homology score, for (a) human (source) - yeast (target) and (b) mouse (source) - yeast (target) comparison.

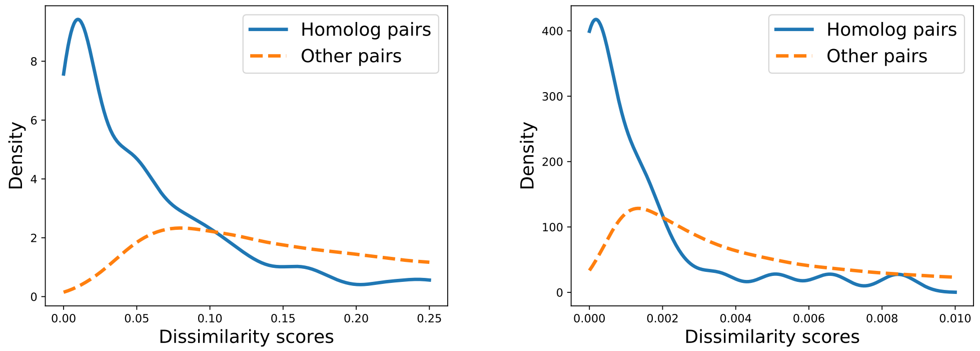

(a) (b)

Figure S2. Distribution of MUNK dissimilarity scores for homologs, compared to distribution for all protein pairs, for (a) human (source) - yeast (target) and (b) mouse (source) - yeast (target) comparison.

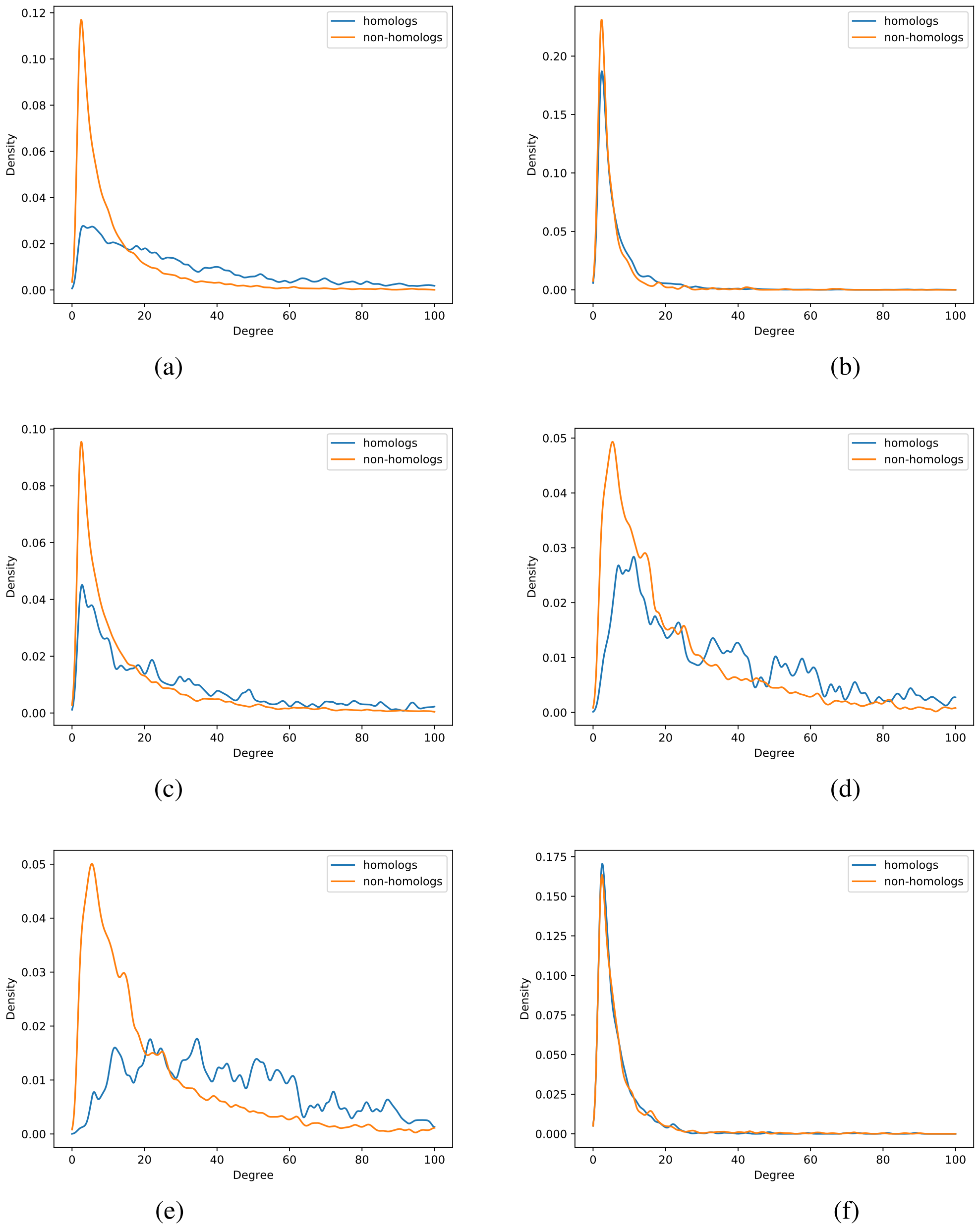

Figure S3. Distribution of node degrees of homologs compared to distribution of non-homologs, for (a) human genes with and without mouse homologs, (b) mouse genes with and without human homologs, (c) human genes with and without baker's yeast homologs, (d) baker's yeast genes with and without human homologs, (e) baker's yeast genes with and without fission yeast homologs, (f) fission yeast genes with and without baker's yeast homologs.

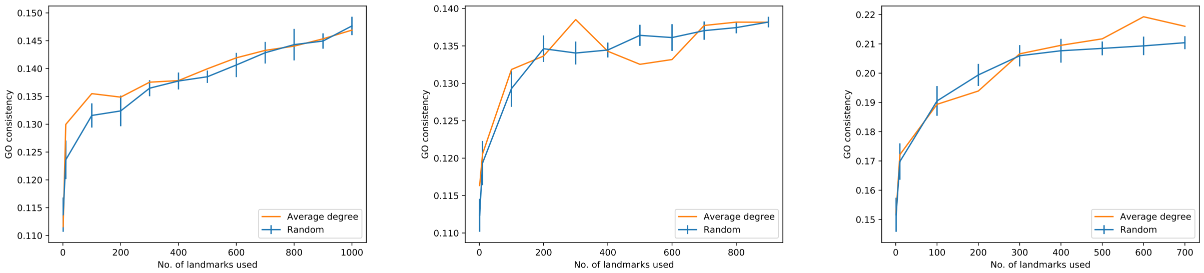

(a) (b) (c)

Figure S4. GO consistency scores of network alignments using MUNK-similarity scores (landmark pairs found in the matching are excluded from GOC score computation) using varying number of MUNK-landmark pairs, for (a) human (source) - mouse (target), (b) human (source) - baker's yeast (target), and (c) baker's yeast (source) - fission yeast (target).

REFERENCES

1. McGary, K. L., Park, T., Woods, J. O., Cha, H., Wallingford, J. B., and Marcotte, E. M. (2010) Systematic discovery of nonobvious human disease models through orthologous phenotypes. *Proceedings of the National Academy of Sciences,* **107**, 6544–6549.
2. Chatraryamontri, A., Oughtred, R., Boucher, L., Rust, J., Chang, C., et al. (2017) The BioGRID interaction database: 2017 update. *Nucleic Acids Research,* **45**, D369–D379.
3. Franceschini, A., Szklarczyk, D., Frankild, S., Kuhn, M., Simonovic, M., Roth, A., Lin, J., Minguez, P., Bork, P., von Mering, C., and Jensen, L. J. (2013) STRING v9.1: protein-protein interaction networks, with increased coverage and integration. *Nucleic Acids Research,* **41**, D808–D815.
4. Collins, S. R., Miller, K. M., Maas, N. L., Roguev, A., Fillingham, J., et al. (2007) Functional dissection of protein complexes involved in yeast chromosome biology using a genetic interaction map. *Nature,* **446**, 806–810.
5. Roguev, A., Bandyopadhyay, S., Zofall, M., Zhang, K., Fischer, T., et al. (2008) Conservation and Rewiring of Functional Modules Revealed by an Epistasis Map in Fission Yeast. *Science,* **322**, 405–410.
6. The UniProt Consortium (2017) UniProt: the universal protein knowledgebase. *Nucleic Acids Research,* **45**, D158–D169.
7. Jacunski, A., Dixon, S. J., and Tatonetti, N. P. (2015) Connectivity Homology Enables Inter-Species Network Models of Synthetic Lethality. *PLOS Computational Biology,* **11**, e1004506.
8. Park, Y. and Marcotte, E. M. (2012) Flaws in evaluation schemes for pair-input computational predictions. *Nature Methods,* **9**, 1134–1136.
9. Hashemifar, S. and Xu, J. (2014) Hubalign: an accurate and efficient method for global alignment of protein–protein interaction networks. *Bioinformatics,* **30**, i438–i444.
